# Pre-hospital management of penetrating neck injuries: derivation of an algorithm through a National Modified Delphi

**DOI:** 10.1186/s13049-024-01291-1

**Published:** 2024-12-02

**Authors:** Christopher Simpson, Harriet Tucker, Joanne Griggs, Maja Gavrilovski, Richard Lyon, Anthony Hudson, Air Ambulance Charity Kent Surrey Sussex, John Breeze, Michael Hughes, Caroline Leech, Adam Watts, Matt Omeara, Cosmo Scurr, Alan Cowley, Ewoud ter Avest, Vicki Brown, Malcolm Russell, Ed Barnard, Phil Cowburn, Tom Hurst, Andy Dunne, Jim Walmsley, Fionna Moore, Will Charlton, Simon Lewis, Pam Hardy, Tim Edwards, James Yates, Nigel Lang, Gordon Ingram, Steve Bell, Clare Fitchett, Andy Curran, Matthew Boylan, Emir Battaloglu, Tim Nutbeam, Matt Taylor, Carl Smith, Erica Ley, Alex James

**Affiliations:** 1 Air Ambulance Charity Kent Surrey Sussex, Hanger 10 Redhill Aerodrome, Redhill, South Nutfield, Surrey, RH1 5YP UK; 2 St Georges Hospital, Blackshaw Road, Tooting, London, SW17 0QT UK; 3 Faculty of Health Sciences, University of Surrey, Guildford, Surrey GU2 7XH UK; 4 St Thomas’ Hospital, Westminster Bridge Road, London, SE1 7EH UK; 5 PHOTON, Pre-Hospital Trainee Operated Research Network, London, UK

**Keywords:** Penetrating neck injury, Trauma, Pre-hospital, Pre-hospital emergency medicine, Helicopter emergency medical services

## Abstract

**Background:**

Timely and effective pre-hospital management of penetrating neck injuries (PNI) is critical to improve patient outcomes. Pre-hospital interventions in patients with PNI can be especially challenging due to the anatomical injury site coupled with a resource-limited environment. Nationally, in the United Kingdom, no consensus statement or expert agreed guidance exists on how to best manage PNI in the pre-hospital setting.

**Method:**

We conducted a national modified e-Delphi study with subject matter experts (SMEs) from multiple professional specialities with experience in the management of PNI. Pre-identified SMEs were contacted and consented prior to participation allowing for a remotely conducted Delphi using REDCap and Microsoft Teams. In Round 1, statements drawn from the literature base were distributed to all SMEs. Round 2 comprised a facilitated and structured discussion of the statements and then an online survey provided final ratification in Round 3. Of the participating SMEs consensus was set a priori at 70%.

**Results:**

Of the 67 pre-identified SMEs, 28 participated, resulting in a response rate of 42%. From the first two rounds, 19 statements were derived with every statement achieving consensus in Round 3. Subsequently, an algorithm for the pre-hospital management of PNI was developed and agreed with SME consensus.

**Conclusion:**

Curation of national consensus statements from SMEs aims to provide principles and guidance for PNI management in a complicated patient group where pre-hospital evidence is lacking. Multi-professional national consensus on the best approach to manage these injuries alongside a novel PNI management algorithm aims to optimise time critical care and by extension improve patient outcomes.

**Supplementary Information:**

The online version contains supplementary material available at 10.1186/s13049-024-01291-1.

## Background

Penetrating neck injury (PNI) is defined as any trauma to the neck that violates the platysma muscle layer [1]. This injury is relatively rare but is a challenging clinical presentation due to the anatomical structures which may be injured. PNI account for up to 1% of all trauma patients with an associated mortality of 3–6% [2]. Pre-hospital teams are often requested to attend patients with PNI due to the nature of the injury and likelihood of clinical deterioration during conveyance to definitive hospital care [3].

Optimal management of PNI in a timely manner is vital to good clinical outcomes, however, management of these injuries is especially challenging within the resource-limited pre-hospital environment. Injuries can span many anatomical structures and physiological systems. Airway insufficiency may result from direct tracheal or external compromise. Ventilatory compromise may result from wounds extending into the thoracic cavity. Haemorrhage from underlying vascular structures can be non-compressible, challenging to control, necessitate advanced resuscitative interventions, and result in significant circulatory and potentially neurological compromise. Similarly, direct spinal cord injury may co-exist or be an isolated injury. In the main, these patients benefit from expeditious transport to a specialist trauma centre. However, the presence of medical professionals, from the first responder to advanced critical care teams, enables clinicians to provide pre-hospital interventions [4]. These may include application of trauma or haemostatic dressings, haemorrhage control or blood product transfusions. Being able to provide interventions at the scene of the incident in a time sensitive manner is important as evidenced within international guidance [1].

A recent review of the management of patients with PNI identified no consensus statements or agreed guidelines on the management of these injuries in the pre-hospital setting in the UK [5], despite those evident through the Western Trauma Association in the US. Although scoping in nature the pre-identified relevant literature from this review formed the basis of a proposed set of management principles and interventions used to guide this study. Consensus statements and guidance are important to all clinicians with varying scopes of practice, to allow for standardised management algorithms within the pre-hospital setting.

We designed a modified e-Delphi study to develop multi-professional national (within the United Kingdom) consensus on the pre-hospital management of PNI. In addition, we aimed to provide a pre-hospital management algorithm based on these core principles to optimise time-sensitive interventions in this challenging patient group.

## Methods

### Study design

The study was initiated, designed and conducted between 1 July 2023 and 1 December 2023 at Air Ambulance Charity Kent Surrey Sussex (KSS). The research group was formed with clinicians from a variety of specialities who are both currently employed within, and have clinical exposure in pre-hospital care.

The modified Delphi method is a structured and iterative approach in achieving consensus to address a particular problem or issue [6]. Typically this comprises identification of an expert panel, repeated rounds of data collection and structured feedback to participants following each round [7, 8]. All participants consented to the study protocol and confidentiality was maintained as far as possible throughout each structured round. The study has been reported following the recommended standards for a Delphi method, as set out by Conducting and Reporting Delphi Studies (CREDES) criteria to allow for improved rigour and transparency in both the conduct and reporting of studies employing the Delphi method [9]. Although, the researchers note the limiting nature in asserting clinical practice guidelines from statements not validated through methods such as Grading of Recommendations, Assessment, Development and Evaluation (GRADE), it was felt that due to the paucity of evidence in this area of clinical practice an expert consensus approach was justified and as such a modified Delphi appropriate [6].

### Participant recruitment

An initial research group was formed (CS, JG, HT, MG, AH). To prevent bias, no members of the research group participated in the Delphi process. The research group identified individuals who were relevant subject matter experts (SMEs) from within the UK. A national, rather than international approach was taken in the first instance to develop consensus and guidelines based on, and directed at, national systems and scopes of practice; these could then be shared on an international stage to agree international practice consensus. These SMEs represented a broad range of clinical backgrounds including Paramedics, Advanced Paramedics, Critical Care Paramedics and Doctors within specialties including Emergency Medicine, Intensive Care Medicine, Anaesthetics, and relevant Surgical specialties. Due to the nuanced study question, the research group adopted this unique approach as opposed to wider advertisement through broader pre-hospital channels.

In addition, the Medical Director and Lead Paramedic of each Air Ambulance organisation along with the Medical Directors of two regional ambulance services were included in the initial invitation to participate. Those who expressed an interest were encouraged to share details of the study with other eligible clinicians, whom may have expertise in the pathology therefore using a snowballing technique [7]. This process ensured a broad range of geographical representation across the UK including both urban and rural regions, and SMEs with both military and civilian pre-hospital clinical exposure, and expertise from a range of specialties. SMEs were invited to participate in all rounds.

### Delphi process employed

**Fig. 1:**
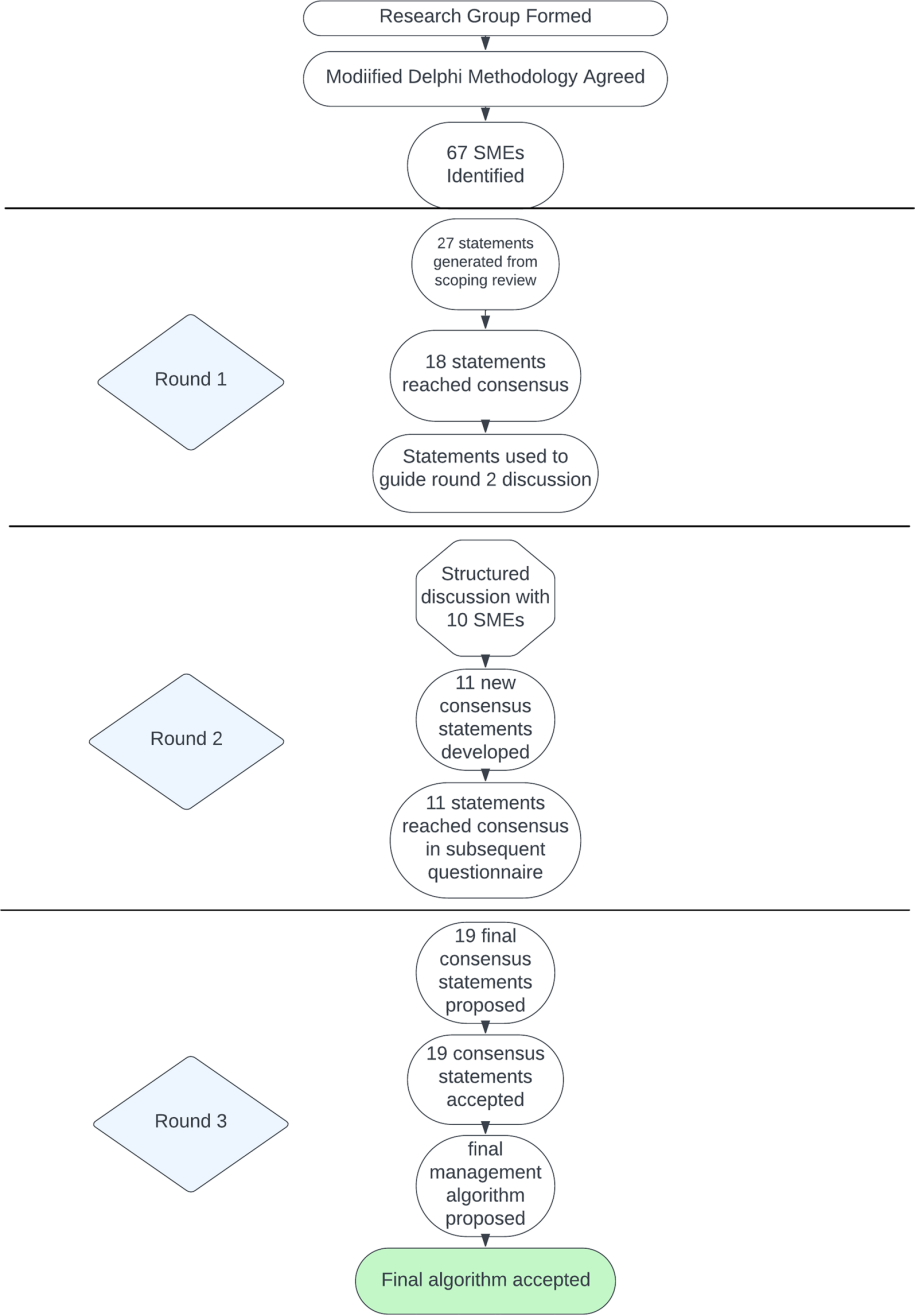
Modified Delphi methodology employed through the study

The modified Delphi process was conducted in three rounds (Fig. 1) to establish both consensus and validity of the proposed statements. Validity in this context evaluated whether statements were clinically relevant, scientifically sound, and practically applicable for developing robust clinical practice guidelines. Each round was designed a priori, and the study design did not change from the original protocol. Identified SMEs (67 total) were provided details of the Delphi study along with a link to the first round on REDCap. Responses to each round were anonymised between SMEs.

Consensus was set a priori at 70% agreement of participating SMEs, in-line with previous Delphi studies [10, 11]. SMEs rated each statements validity with regard to practical applicability in the clinical setting. Free text feedback was optional for each statement and responses to this were used to guide the structured discussion in Round 2 and generation of new potential statements for consensus. This iterative process allowed for refinement of statements to enhance their validity while maintaining their clinical utility.

Rigour and transparency throughout the study procedures was ensured by the research group. The surveys delivered in Round 1 and Round 3 used the software REDCap (Research Electronic Data Capture) hosted at KSS. REDCap is a survey tool that is General Data Protection Regulation (GDPR) compliant and certified to ISO 27001 standard [12]. Following data collection anonymised data was exported to Excel (Version 16.79.1). The systematic approach to data collection and analysis strengthened the methodological validity of the consensus-building process.

### Round 1: Initial statement consensus

In Round 1, statements for consideration were generated by the research group from the scoping review and evidence base summarised in the aforementioned review paper [5]. Accompanying information that provides further information on each statement was provided to contextualise and situate each statement with supporting evidence. Respondents were asked to score the statements using a Likert scale from 0 to 10 (0 indicating strongly disagree and 10 indicating strongly agree). The wording of each statement was agreed between the co-authors prior to Round 1. Round 1 was undertaken in July and August 2023.

### Round 2: Structured facilitated discussion

Round 2 consisted first of an online structured discussion between SMEs, chaired by one research group member (AH), and moderated by the other research group members. All identified SMEs were invited to this discussion and consent was ensured from all SMEs prior to commencement. During this structured discussion, both accepted and rejected statements from Round 1 were discussed along with the anonymised comments submitted pertaining to each statement. During this structured discussion, two research group members took detailed notes (JG/MG) and all comments made in Microsoft Teams chat were captured. From these, the research team made one of three decisions from the Round 1 statements:

- Accepted verbatim and taken through to Round 3
- Discarded and not taken through to subsequent rounds
- New statement generated as a collapsed/amalgamated/revised version of Round 1 statement.

All new statements generated through option C were then taken back to the panel of SMEs involved in Round 2 for approval, prior to being advanced through to Round 3.The new statements generated in Round 2 are shown in Appendix 1 in supplementary material. Round 2 was completed on 7 September 2023.

### Round 3: Final ratification

Round 3 took the statements generated from Round 1 and 2 back to the SME group for final ratification. From the questions, statements, and comments in Round 1, alongside the structured discussion in Round 2; a new proposed management algorithm was generated by the research group. At this point the algorithm was communicated to the SMEs. Round 3 was undertaken in November and December 2023.

### Patient and Public Involvement

Lay representation on the Charity Board at KSS expressed support for continued research into the management of pre-hospital traumatic injuries, including those with penetrating neck trauma. Patients were not directly involved in the study design, recruitment, or conduct. Results and clinical interpretation of the study will be shared with both lay representatives and partner organisations as deemed appropriate.

### Ethical considerations

The Health Research Authority (HRA) Medical Research Council decision tool was used to establish the need for Research Ethical Committee (REC) approval. The toolkit advised that REC approval would not be required. Prior to commencing the study, the KSS Research and Innovation Committee approved the protocol and additional research materials.

## Results

### Summary of each round

In Round 1, 28 of the 67 initially identified SMEs responded to the survey giving a response rate of 42%. The profession, background and service of these SMEs was collected to describe the geographical and experiential expertise contributing to the Delphi (Table 1). In Round 1, 27 statements were considered, of which 18 reached consensus. Three Yes/No questions and one priorities question were also used to inform the management algorithm. One question on time-intervals was also included in Round 1, but this topic was taken no further following the structured discussion as it was deemed outside the scope of this project.

**Table 1:** Participant demographics, background, professional specialty and individual PHEM representation amongst the SMEs participating in Round 1

		Respondents
Profession	Medic	14
	ACP/CCP	9
	Consultant Paramedic	1
Medical specialty	Anaesthetics	3
	Emergency Medicine	9
	GP/PHEM	2
	Intensive Care Medicine	2
	Oral and Maxillofacial surgery	1
	Trauma surgery	1
PHEM service representation	Air Ambulance Charity Kent Surrey Sussex	5
	Devon Air Ambulance Trust	3
	Dorset and Somerset Air Ambulance Trust	1
	East Anglia Air Ambulance	2
	Emergency Medical Retrieval Service	1
	Emergency Medical Retrieval Service	1
	Great Western Air Ambulance Charity	1
	Hampshire and Isle of Wight Air Ambulance	2
	London Air Ambulance Charity	2
	Lincolnshire and Nottinghamshire Air Ambulance	1
	MAGPAS	1
	North-West Air Ambulance Trust	2
	South-East Coast Ambulance Trust	1
	South-West Ambulance Service Foundation Trust	1
	The Air Ambulance Service	1
	West Midlands MERIT	1
	No current PHEM service	2

PHEM; Pre-hospital Emergency Medical Service; SMEs, Subject Matter Experts; ACP, Advanced Clinical Practitioner; CCP, Critical Care Paramedic; GP, General Practitioner. MERIT; Medical Emergency Response Incident Team

Round 2, in which 10 SMEs took part started with a facilitated structured discussion. From this event a further 11 consensus statements were generated by the research group. These statements were sent out to the SMEs who had participated in the structured discussion and all 11 developed statements reached consensus and were thus taken through to Round 3.

Round 3 included 25 of the initially identified SMEs responded to this final round, giving a response rate of 37%. The profession and background of these SMEs is summarised in Table 2. 19 final consensus statements generated from the previous two rounds were proposed and all 19 reached consensus (Table 3).

**Table 2:** Participants demographics, background and professional specialty providing final ratification to statements and algorithm in Round 3

Profession	Medic	14
	ACP/CCP	9
	Consultant Paramedic	2
Medical Specialty	Anaesthetics	2
	Emergency Medicine	8
	GP/PHEM	1
	Intensive Care Medicine	1
	Trauma surgery	2

ACP, Advanced Clinical Practitioner; CCP, Critical Care Paramedic; GP, General Practitioner; PHEM, Pre-hospital Emergency Medicine

**Table 3:** Consensus statements accepted in Round 3

Catastrophic haemorrhage	Consensus
Packing of wounds with haemostatic gauze (when possible) and application of direct pressure represents the mainstay of treatment	24/25 (96%)
In the context of haemorrhage control, consider temporary rapid wound closure with, for example, staples, sutures or a novel wound closure device, subject to available skill set, equipment and expertise	22/25 (88%)
In the context of haemorrhage control, consider haemorrhage control using balloon catheter tamponade with a catheter device. Examples may include a urinary Foley © catheter, Epistat^©^ or RapidRhino^©^	23/25 (92%)
Once haemostasis has been achieved the dressing or other device should not be removed until a place of safety is reached i.e. hospital with appropriate surgical skills to control haemorrhage	22/25 (88%)
*Airway and ventilation*	
Clinicians should be mindful that advanced interventions, including PHEA, may not always be necessary and/or available. If haemorrhage control is achieved or available interventions optimised, clinicians should not delay scene time if onward transfer to an appropriate centre is clinically and logistically appropriate	23/25 (92%)
Where relevant expertise is available and a specific indication for PHEA exists, this intervention should be undertaken expediently. Where this is not present, consider rapid transfer to receiving hospital with ongoing spontaneous ventilation	24/25 (96%)
Where skills are available and indications are present, in most cases drug assisted PHEA using a rapid sequence technique should be considered as the first attempted airway intervention	24/25 (96%)
Cricoid pressure should be avoided	22/25 (88%)
Choice of laryngoscopy device should be guided by experience and personal preference	20/25 (80%)
Front of neck access should be attempted in a cannot intubate cannot ventilate scenario	24/25 (96%)
If a definitive airway cannot be achieved through RSI, direct intubation of a transected trachea should be considered if applicable	23/25 (92%)
In the context of PNI, clinicians should be aware of the potential for intrathoracic injury and treat in accordance with clinical assessment and accepted guidelines	25/25 (100%)
Clinicians should be aware of the risks of PPV in patients with a known or possible airway injury due to the risk of surgical emphysema worsening the clinical condition	25/25 (100%)
Clinicians should be aware of the risk of false passage and ETT misplacement with bougie use in the context of occult or clinically evident tracheal injury, but this should not discourage from its routine use	25/25 (100%)
*Resuscitation measures*	
Clinicians may consider undertaking resuscitative thoracotomy in injuries in close proximity to the clavicle, if it can be undertaken within an appropriate timeframe and would provide specific patient benefit. This includes relieving cardiac tamponade, direct haemorrhage control or allowing aortic compression	24/25 (96%)
In patients who have sustained cardiac arrest from PNI, immediate haemorrhage control is the priority. Following this, interventions specific to the patient’s clinical presentation should be undertaken concurrently whenever possible. This may include airway management, vascular access and appropriate volume resuscitation, chest decompression and RT where appropriate	24/25 (96%)
*Disability prevention*	
Clinicians should undertake careful patient handling, positioning, and packaging; consider appropriate aspects of cervical spine control, in line with local guidelines and injury pattern, balanced with the need for ongoing assessment and treatment of the penetrating neck injury	21/25 (84%)
Cervical collars should be avoided, and spinal immobilisation achieved through the use of alternative methods such as head blocks whenever possible	24/25 (96%)
Cervical spine control should be reserved for patients with hard neurological signs	20/25 (80%)
Algorithm	20/25 (80%)

PHEA, Pre-hospital emergency anaesthesia; RSI, rapid sequence intubation; PNI, penetrating neck injury; PPV, positive pressure ventilation; ETT, endotracheal tube

**Fig. 2:**
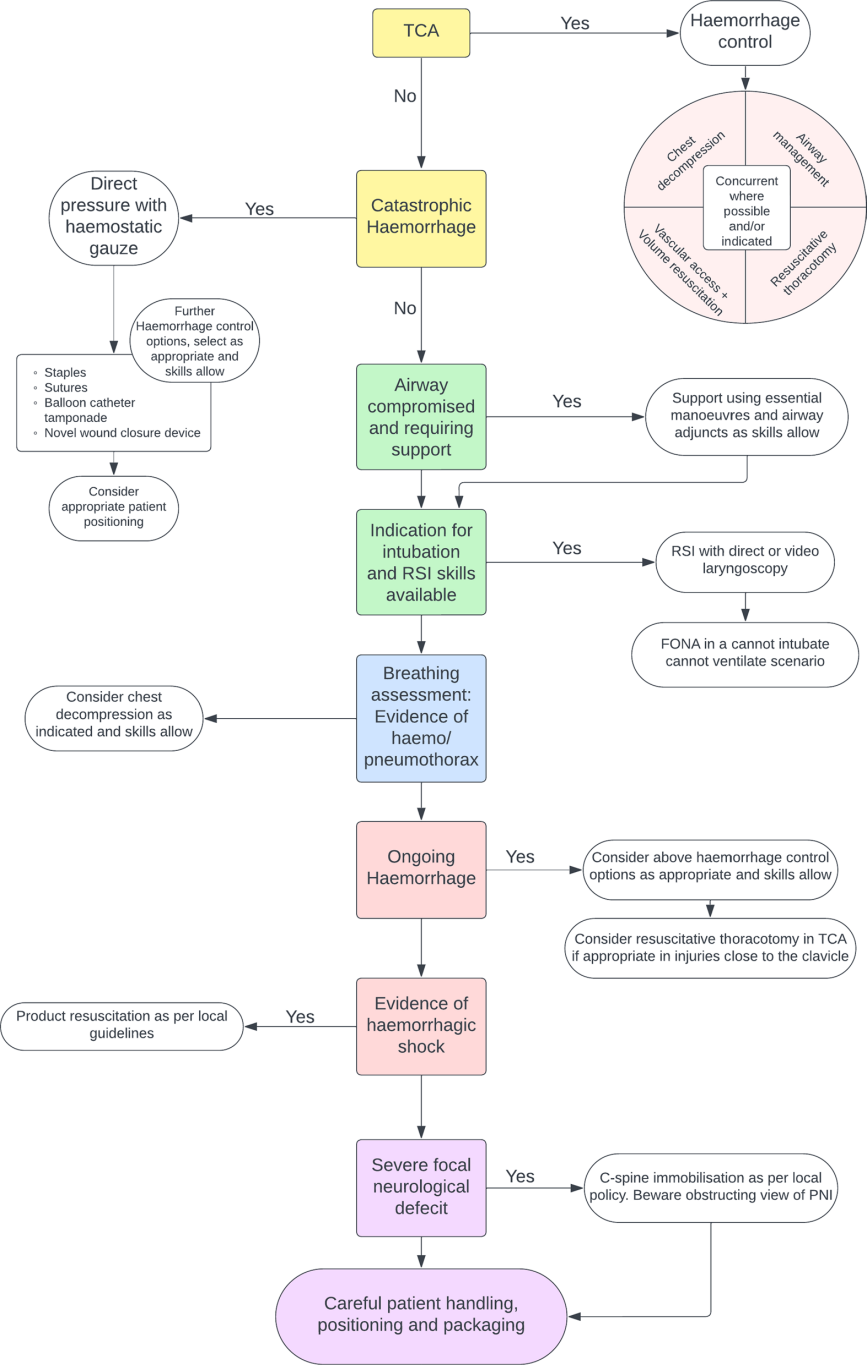
Resultant algorithm for management of PNI

A final management algorithm was proposed which also reached consensus for acceptance. The proposed management algorithm was developed following the consensus statements that were agreed by the SME group, along with the discussion generated in Round 2. The final algorithm was ratified for publication in Round 3 with 80% agreement from responding SMEs (Fig. 2).

## Discussion

The agreed consensus statements from this Delphi will provide guidance for management of a complicated and nuanced patient group with PNI where pre-hospital evidence is currently lacking. Overarching statements were curated to provide principles of management that are applicable to all levels of pre-hospital practitioner, with the understanding that certain interventions will not be within the scope of practice of all. It is hoped that the publication of the PNI Delphi will drive both National and International discussion to reach consensus on the pre-hospital management of PNI, which incorporates pre-hospital specialists to encourage decisions on profession specific interventions.

We note that in Round 1, SMEs were more likely to reach consensus where broad principles of management were suggested as opposed to specific treatment recommendations, potentially with specific equipment that operators may not be familiar with. This approach was thus used to develop the statements proposed in Round 2, and the final agreed upon statements. However, further detailed discussions may be required to facilitate more specific treatment recommendations. This was reflected within the wording choices first adopted by the research team, as each round iterated the language softened to reflect the uncertainty and context specific alterations to each statement.

This study was conducted along the guidelines set out by the aforementioned CREDES study [9]. SME selection was performed by the research group based on knowledge of SMEs with experience managing this specific patient population. There was the potential for selection bias to be introduced through this process so the step to invite the Medical Directors and Lead Paramedic of each service (or their nominated representative) was introduced to mitigate against this and ensure a broad consensus from a range of stakeholders. The SMEs demonstrated an appropriate level of engagement with the study which was maintained throughout. As expected, Round 2 required a greater and more specific time input and had the lowest response rate, however the dissemination of the outcomes of Round 2 back to the whole SME group in Round 3 was conducted to remove any self-selection bias potentially incurred because of this. Nevertheless, the authors recognise the potential for some selection bias. In addition, all identified SMEs are from UK based pre-hospital practice, thus potentially limiting the external validity to countries with varying pre-hospital systems.

### Catastrophic haemorrhage

The SME group agreed that direct pressure, along with packing of wounds with haemostatic gauze where possible, represents the mainstay of treatment for catastrophic haemorrhage in this junctional region. This is in agreement with the published literature which reports haemostasis to be achieved in between 67 and 100% of cases with this method [13].

Augmentation of wound closure and cessation of haemorrhage was a significant topic of discussion between the SMEs in Round 2, following an inability to reach consensus in Round 1. It was felt that multiple options of method, device and technique were available with none showing proven superiority, albeit with each demonstrating individual proven success [14–17]. As such the consensus statement advocates for the use of a method and/or device appropriate to both that patient, situation, and operator. All SMEs agreed throughout that once haemorrhage control had been achieved, the method of control should not be removed until a location able to provide definitive care had been reached.

### Airway and ventilation

The SME group were keen during the facilitated discussion, with subsequent ratification in Round 3, to ensure certain key logistical points were made. The clear theme to these two first statements was to ensure that discussion of management principles did not delay ground emergency medical teams waiting for pre-hospital teams that can provide advanced interventions, when it may be more appropriate to start moving towards an appropriate receiving facility to receive definitive care.

The recommendation for drug assisted Pre-hospital Emergency Anaesthetic (PHEA) as the first attempted airway intervention, where appropriate, has limited published evidence in the pre-hospital setting. However in-hospital case series with up to 100% success rate have been reported [18] and a review of the topic concluded Rapid Sequence Induction (RSI) to be the preferred method of intubation in this patient group [2]. In-line with the Difficult Airway Society (DAS) guidelines 2015 [19], the SMEs agreed that Front of Neck Access (FONA) should be attempted in a cannot intubate cannot oxygenate scenario. The evidence for the success of this procedure in the pre-hospital setting supports this, with success rates ranging from 82 to 97% having been reported [20–22].

There was unanimous SME agreement that the potential for thoracic injury must be considered. This is consistent with a previously reported case series of PNI patients, within which 2% of isolated neck injuries also required chest decompression [23]. The SMEs were also in unanimous agreement to highlight the potential complications with both the use of a Bougie™ and Positive Pressure Ventilation (PPV) in the context of PNI, however these risks should not detract from their use where appropriate.

### Resuscitation measures

The SME group were keen that this Delphi of PNI management did not extend beyond its initial scope. Therefore decisions regarding interventions such as Resuscitative Thoracotomy (RT), and others indicated in the management of traumatic cardiac arrest, should remain within the remit of local service guidance, supported by national recommendation [24, 25]. However, the SMEs were in agreement with published US guidance for in-hospital practice [26] that RT may be an appropriate pre-hospital intervention if used in an appropriate time frame, targeting a specific suspected pathology, with appropriate patient selection, with the caveat that this guideline focuses on in-hospital rather than pre-hospital practice, again highlighting the lack of pre-hospital guidance which this project is aiming to address.

In PNI patients who have sustained a traumatic cardiac arrest, the SME group felt that haemorrhage control is the primary priority, but interventions could not be given in a pre-determined order. As such a vortex model was favoured with clinicians aiming to address priorities in an order appropriate to that patient’s presentation, concurrently where possible.

### Disability prevention

The SME group felt that generic recommendations regarding c-spine management were outside the scope of this Delphi project. As such local guidance should be followed regarding cervical spine control.

Rates of unstable injury are as low as less than 1% in non-ballistic penetrating neck injury [27] and cervical spine immobilisation increases the odds ratio for death to 2.06 (95% CI 1.35–3.13) in penetrating neck trauma [28]. More specifically in a small case series of PNI, c-spine collars were concluded to have the potential to hide signs of life-threatening conditions, including tracheal deviation, subcutaneous emphysema, large expanding haematoma, and diminished or absent carotid pulses [29]. In-line with these findings the SME group reached consensus on the avoidance of c-spine collars whenever possible and on only applying cervical spine control in the presence of hard neurological signs of injury. This is in line with other recent clinical guidelines on this topic [30].

### Limitations

The proposed set of interventions and management principles in Round 1 were the result of the literature reported in the referenced scoping review [5]. Due to the limited nature of the evidence surrounding this question this was deemed appropriate to inform the first stage of the Delphi process, however, the researchers recognise the methodological limitations of using a scoping review as opposed to one that is systematic in nature [31]. Overall, the researchers regarded the rigorous and transparent conduct of the scoping review to be sufficient to identify any knowledge gaps in an area lacking in evidence, although the lack of systematic review is clearly a limitation to be acknowledged. A second limitation relates to the wording of each statement, as these were iterated between each round the precise wording ‘softened’ from the term ‘should’ to ‘consider’ etc. Other wording such as ‘might’ may be adopted in future studies to acknowledge the uncertainty within this area of clinical practice in-line with recommendations from the SMEs.

## Management algorithm

The algorithm is wholly designed to form a framework from which to approach this difficult patient group. We assert that the algorithm is *not* designed to form a checklist that must be worked through in a systematic manner. By virtue of this complex patient group, interventions should be nuanced on a patient-by-patient basis. The development of a checklist type algorithm is not attainable nor appropriate in PNI management. Certain interventions mentioned at certain points in the algorithm e.g. resuscitative thoracotomy may not be appropriate at that point or in that patient. As such it is paramount that clinicians use their experience and judgement alongside the proposed framework. This methodology follows that shown in previous modified Delphi studies [32].

## Conclusion

This study has generated a series of consensus statements and a management algorithm that clinicians may use to guide their practice in management of pre-hospital patients with a PNI. This is a complex patient group requiring nuanced care, particularly within the resource limited pre-hospital setting. These consensus statements and algorithm could form the foundation of management, from which clinicians can apply those elements applicable to each patient on case-by-case basis within an individual’s pre-hospital scope of practice.

## Supplementary Information

Supplementary file 1

## Data Availability

Where deemed appropriate data is available from the corresponding author upon reasonable request.

## Abbreviations

HEMS: Helicopter Emergency Medical Services
PHEM: Pre-hospital Emergency Medicine
PNI: Penetrating Neck Injury
RSI: Rapid Sequence Intubation
PHEA: Pre-hospital Emergency Anaesthesia
UK: United Kingdom
ACP: Advanced Clinical Practitioner
CCP: Critical Care Paramedic
GP: General Practitioner
PPV: Positive Pressure Ventilation
ETT: Endotracheal Intubation
SME: Subject Matter Expert
CREDES: Conducting and Reporting Delphi Studies
KSS: Air Ambulance Charity Kent Surrey Sussex
REDCap: Research and Electronic Data Capture
GDPR: General Data Protection Regulation
FONA: Front of Neck Access
UK: United Kingdom
DAS: Difficult Airway Society
TCA: Traumatic Cardiac Arrest
