# Supplementary material for: Pre-hospital management of penetrating neck injuries: derivation of an algorithm through a National Modified Delphi
Source: Scand J Trauma Resusc Emerg Med. 2024 Dec 2;32:123. doi: 10.1186/s13049-024-01291-1 (PMC11613838; doi:10.1186/s13049-024-01291-1)
Supplement: Supplementary file 1 [file 13049_2024_1291_MOESM1_ESM.pdf]

Penetrating Neck Injury Delphi

Please complete the survey below, thank you.

Thank you for taking part in this National Delphi project.

De-identified results comprising overall scores for each item and a narrative summary of findings, comments and suggestions will be sent to each panel member after the first round.

The authorship team anticipates a total of three rounds. The first few questions relate to your background and experience.

Name ______________________________

Grade ______________________________

Base Specialty ______________________________

Service ______________________________

Regarding the quality of the evidence base, with respect to the initial paper scoping penetrating neck injuries. Please consider the following questions.

Simpson, C., Tucker, H., & Hudson, A. Pre-hospital management of penetrating neck injuries: a scoping review of current evidence and guidance. Scandinavian Journal of Trauma Resuscitation Emergency Medicine 29, 137 (2021).

Simpson *et al.*
Scand J Trauma Resusc Emerg Med (2021) 29:137
https://doi.org/10.1186/s13049-021-00949-4

Scandinavian Journal of Trauma, Resuscitation and Emergency Medicine

REVIEW

Open Access

Pre-hospital management of penetrating neck injuries: a scoping review of current evidence and guidance

Christopher Simpson[1*], Harriet Tucker[1,2] and Anthony Hudson[1,2]

Abstract

Penetrating injuries to the neck pose a unique challenge to clinicians due to the proximity of multiple significant anatomical structures with little protective soft tissue coverage. Injuries to this area, whilst low in incidence, are potentially devastating. Respiratory, vascular, gastro-oesophageal and neurological structures may all be involved, either in isolation or combination. These injuries are particularly difficult to manage in the resource poor, often austere and/or remote, pre-hospital environment. A systematic scoping review of the literature was conducted to evaluate the current available research pertaining to managing this injury profile, prior to the patient arriving in the emergency department. The available research is discussed in sections based on the commonly used trauma management acronym 'cABCD' (catastrophic haemorrhage, Airway, Breathing, Circulation, Disability) to facilitate a systematic approach and clinical evaluation familiar to clinicians. Based on the available reviewed evidence, we have proposed a management algorithm for this cohort of patients. From this we plan to instigate a Delphi process to develop a consensus statement on the pre-hospital management of this challenging presentation.

Keywords: Penetrating neck injury, Penetrating trauma, Stabbing, Pre-hospital, Immediate

Background

Penetrating neck injury (PNI) is defined as any trauma to the neck that violates the platysma muscle layer. Penetrating injuries to the neck pose a unique challenge to clinicians, especially within the resource-limited pre-hospital environment. Optimal management within this immediate time critical setting is paramount to good clinical outcomes. Previous studies have suggested these injuries account for up to 1% of all trauma patients with an associated mortality ranging from 3 to 6% [1].

The majority of recent research has focused on the management of these injuries on arrival to the emergency department. From this, recommendations and guidance have been implemented to guide decision-making regarding surgical exploration and the use of computed tomographic angiography [2].

At the time of writing, there exists currently no consensus statement or agreed guidelines regarding the pre-hospital management of penetrating neck injuries that these authors are aware of. The aim of this review is therefore to evaluate current practice and collate the current evidence for the management of PNIs before the patient reaches the emergency department. These management principles may then be used to establish a standard of care for use by the pre-hospital clinical team attending to the patient, within both their scope of practice and their current service guidelines.

*Correspondence: Christopher.simpson@doctors.org.uk
[1] Emergency Department, St. George's Hospital Trust, Blackshaw Rd., Tooting, London SW17 0QT, UK
Full list of author information is available at the end of the article

© The Author(s) 2021. **Open Access** This article is licensed under a Creative Commons Attribution 4.0 International License, which permits use, sharing, adaptation, distribution and reproduction in any medium or format, as long as you give appropriate credit to the original author(s) and the source, provide a link to the Creative Commons licence, and indicate if changes were made. The images or other third party material in this article are included in the article's Creative Commons licence, unless indicated otherwise in a credit line to the material. If material is not included in the article's Creative Commons licence and your intended use is not permitted by statutory regulation or exceeds the permitted use, you will need to obtain permission directly from the copyright holder. To view a copy of this licence, visit http://creativecommons.org/licenses/by/4.0/. The Creative Commons Public Domain Dedication waiver (http://creativecommons.org/publicdomain/zero/1.0/) applies to the data made available in this article, unless otherwise stated in a credit line to the data.

Anatomy

The neck is a region of complex anatomy, with many vital structures located in very close proximity. These structures include airway, vascular, neurological, musculoskeletal and gastrointestinal components. For clinical evaluation, the anterior neck is divided in to three distinct zones. The most commonly accepted classification of these zones is by Roon and Christiansen [3]:

- Zone 1: Extending from the clavicles to the cricoid cartilage.
- Zone 2: Extending from the inferior margin of the cricoid cartilage to the angle of the mandible.
- Zone 3: Extending from the angle of the mandible to the base of the skull.

This classification refers to the anterior triangles of the neck, defined by the anterior border of the sternocleidomastoid muscle, the anterior midline and the mandible. Within this region is where the majority of the crucial structures are located (Table 1). It is important to note, however, that the zone in which an external wound is located does not necessarily correlate to which internal structures are injured; this is a function of both entry location and vector of travel [4]. Recent studies suggest zone 2 is the most commonly injured (38–67%), followed by zone 3 (16–19%), and zone 1 (13–18%) [5–8].

Method

This review did not meet the criteria for prospective registration with PROSPERO and was thus not registered. However, the scoping systematic review methodology was followed throughout. PubMed was used to search the MEDLINE database. Ovid was used to search the Cochrane database of systematic reviews and Embase. All databases were interrogated for any combination of the terms 'stab,' 'stabbing,' 'penetrating,' 'neck,' 'cervical,' 'pre-hospital,' 'prehospital' and 'immediate'. These searches resulted in 278 and 239 results respectively. All primary research and review articles written in English language and published between 1/1/2000 and 31/12/2020 were evaluated. Exclusion criteria were papers not written in English language, those pertaining to blunt trauma, case reports, letters to the editor, opinion papers and papers not directly reporting, or reporting as part of a review, novel experimental data. The remaining papers were then screened for relevance to pre-hospital practice, reviewing each study's location, setting, timing, number of included patients and intervention. A total of 20 articles were identified from the initial database search including nine systematic reviews and 11 original research papers. The reference lists of these included studies and relevant reviews were then screened in the same manner for further relevant publications not captured in our initial search. This elicited a further seven original research papers, to make a total of 27 papers included in the review. The primary research papers from both the initial database search along with the secondary reference search are displayed in Table 2. The search strategy is displayed in the PRISMA diagram (Fig. 1).

Results

Initial assessment

Initial findings on clinical examination are traditionally grouped in to 'hard' and 'soft' signs of injury (Table 3) [2]. Hard signs suggest major airway, vascular or digestive tract injury. These include airway compromise, air bubbling in the wound, active bleeding, an expanding or pulsatile haematoma, shock and neurologic deficit.

Hard signs occur in less than 10% of patients with PNI. However they are highly specific for serious injury, with a positive predictive value of almost 90% for a vascular or aerodigestive tract injury [5].

Catastrophic haemorrhage

Uncontrolled haemorrhage is a major cause of morbidity and mortality in trauma and a large proportion of this mortality occurs in the pre-hospital setting [9]. Neck wounds are amongst those classed as junctional wounds to which tourniquets cannot be applied. Simple gauze dressings and the application of direct pressure were traditionally the mainstay of treatment. More recently

Table 1 Anatomic contents of the anterior zones of the neck. Adapted from Hanlon and Adams [26]

Zone	Contents
1	Major vasculature of superior mediastinum, subclavian vessels, common carotid artery, internal jugular vein, trachea, lung apex, oesophagus, vagus, recurrent laryngeal and phrenic nerves
2	Common, internal and external carotid arteries, jugular veins, trachea, larynx, pharynx, oesophagus, vagus, recurrent laryngeal and phrenic nerves
3	Internal and external carotid arteries, jugular veins, oropharynx, cranial nerves 9–12

Table 2 Primary research articles identified through the search strategy

Year	Study title	Author	Study type	Level of evidence	Patient number	Military/civilian
Catastrophic haemorrhage						
2015	Celox-coated gauze for the treatment of civilian penetrating trauma: a randomised clinical trial	Hatamabadi et al	RCT	1b	160	Civilian
2016	A multi-institutional study of haemostatic gauze and tourniquets in rural civilian trauma	Leonard et al	Case series	4	40	Civilian
2015	Prehospital use of hemostatic dressings by the Israel Defense Forces Medical Corps: a case series of 122 patients	Shina et al	Case series	4	122	Military
Airway, breathing and ventilation						
2010	A meta-analysis of prehospital airway control techniques part II: alternative airway devices and cricothyrotomy success rates	Hubble et al	Meta-analysis	3a	512	Civilian
2000	Emergency airway management in penetrating neck injury	Mandavia et al	Case series	4	82	Civilian
2009	Airway Management of Two Patients with Penetrating Neck Trauma	Bhattacharya et al	Case series	4	2	Civilian
2020	Neck Injuries—Israel defense forces 20 years' experience	Tsur et al	Case series	4	41	Military
2014	Prehospital and en route cricothyrotomy performed in the combat setting: a prospective, multicentre, observational study	Barnard et al	Prospective case series	4	34	Military
Circulation						
2006	Emergency department resuscitative thoracotomy for non-torso injuries	Sheppard et al	Case series	4	27	Civilian
2016	The iTClamp in the management of prehospital haemorrhage	Tan et al	Case series	4	10	Civilian
2019	Management of life-threatening haemorrhage from maxillofacial firearm injuries using Foley catheter balloon tamponade	Jose et al	Case series	4	11	Military
2013	The iTClamp controls junctional bleeding in a lethal swine exsanguination model	Fillips et al	Cohort study	2b	20 animal swine model	N/A
Disability prevention						
2010	Spine immobilisation in penetrating trauma: more harm than good?	Haut et al	Case control	3b	45,284	Civilian
2009	Increased risk of death with cervical spine immobilisation in penetrating cervical trauma	Vanderlan et al	Case control	3b	188	Civilian
2011	Unstable cervical spine fracture after penetrating neck injury: a rare entity in an analysis of 1069 patients	Lustenberger et al	Case series	4	1069	Civilian
2009	Neurologic sequelae of penetrating cervical trauma	Vanderlan et al	Case series	4	196	Civilian
2000	Prehospital stabilization of the cervical spine for penetrating injuries of the neck—is it necessary?	Barkana et al	Case series	4	44	Military
2009	Learning the lessons from conflict: prehospital cervical spine stabilisation following ballistic neck trauma	Ramasamy et al	Case series	4	90	Military

RCT Randomised control trial

however, haemostatic dressings have been developed. These contain agents which enhance blood clotting and promote haemostasis, and have been shown to achieve haemostasis in the prehospital environment in between 67 and 100% of cases, with a median success rate of 90.5% [10].

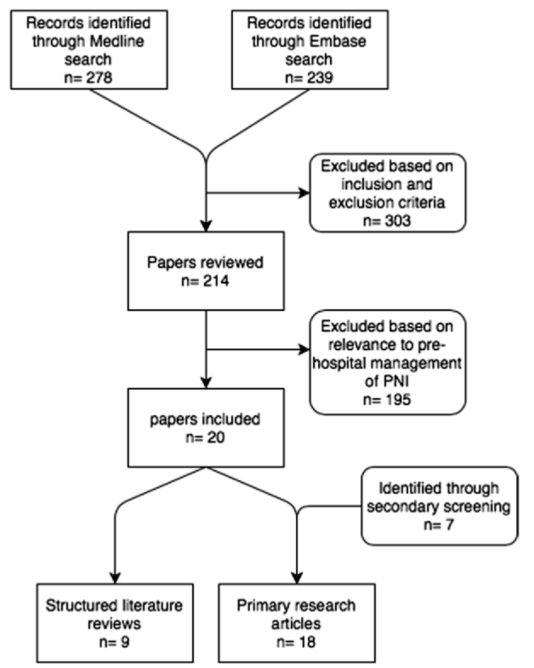

Fig. 1 PRISMA diagram of search strategy

Table 3 Hard and soft signs of injury in penetrating neck injury. Adapted from Sperry et al. [2]

Hard signs	Soft signs (non-exhaustive)
Massive subcutaneous emphysema/ air bubbling through the wound	Proximity of the wound to the carotid artery or jugular vein
Airway compromise	Non-expanding haematoma
Expanding or pulsatile haematoma	Change in voice and/or hoarseness
Active bleeding	Dysphagia
Shock	Upper extremity pulse deficit
Haematemesis/haemoptysis	Palpable crepitus
Neurologic deficit	

Multiple haemostatic dressings are available including QuickClot® Combat Gauze™, favoured by the US military, and Celox™-coated gauze, favoured in the UK. These two are proven to be significantly more likely to arrest haemorrhage than standard gauze alone, being effective in more than 88% of cases and have no associated adverse events [10–14].

In practical terms all products suggest the same use in the context of penetrating neck injuries. The gauze should be packed tightly into the neck wound as able. The remainder of the gauze can then be used over the top of the PNI. Direct pressure should be applied for a minimum of 3 minutes. Following this, a further dressing should be applied over the top of the haemostatic gauze to maintain pressure as able. The gauze should not be removed until the patient is in a place of safety. In PNI, once the immediately life-threatening catastrophic haemorrhage has been controlled, ongoing direct pressure to the neck must be used in balance against the risk of causing cerebral ischaemia from reduced blood flow.

Airway control and ventilation

Airway management in PNI is potentially challenging. The airway may be complicated by the injury itself, the presence of blood or secretions, or may be secondarily distorted by soft tissue swelling or haematoma formation. The bleeding upper airway poses many challenges including preoxygenation and denitrogenation being less efficient, hypovolemia with impending or established circulatory collapse and also human factors including the clinical care team being overwhelmed by stress [15].

Due to the range of potential presentations, there is no consensus on airway management in PNI. As such, decisions on airway control should be taken on a patient-by-patient basis. Indications for intubation include airway compromise/injury, apnoea or respiratory failure, hypoxia and a reduced level of consciousness [16]. Early recognition of any of these indications is crucial in both the pre-hospital setting and in PNI, especially when these two factors are in combination.

Wherever possible, spontaneous ventilation should be maintained in the acute adult airway trauma patient [17]. However, in the time critical agitated patient, the first attempted intervention should be a Rapid Sequence Intubation (RSI), but with certain proposed modifications. These include, where possible, avoidance of both cricoid pressure and intermittent positive pressure ventilation via a facemask or supraglottic airway device.

Rapid sequence intubation

Airway management options are more limited in the pre-hospital environment than in the emergency department due to resource limitations, and research is lacking; however, extrapolation of the available emergency department evidence may reassure pre-hospital clinicians. Mandavia et al. [18] retrospectively analysed all emergency department intubations at a level 1 trauma center over a 3-year period. In this time 748 patients with PNI were evaluated, of which 11% required emergency airway control. Of these 67.2% were intubated through RSI with direct laryngoscopy with a 100% success rate. This finding is in keeping with a previous retrospective review of 107 patients with PNI which demonstrated no difference in success rate when advanced airway techniques, such as

awake fibreoptic intubation, were used compared to RSI and direct laryngoscopy [19].

This has led a recent review to conclude RSI to be the preferred method of intubation for patients with PNI [1]. The available in-hospital data suggests a strong likelihood of success with emergency intubation through RSI and direct laryngoscopy in PNI. Therefore, despite the added difficulties in performing a pre-hospital RSI, clinicians should not be deterred from gaining immediate airway control when there is an immediate need to do so.

The use of video laryngoscopy (VL) is becoming more widespread both within the hospital and pre-hospital environment, expedited by the need for changes in practice in response to COVID19. The potential benefits of video laryngoscopy were summarised in a recent Cochrane review as, 'Video laryngoscopes may reduce the number of failed intubations, particularly among patients presenting with a difficult airway' [20]. No evidence was available comparing video with direct laryngoscopy (DL) in relation to pre-hospital PNI management. Therefore, these reported benefits must be balanced against potential PNI specific difficulties, such as blood obscuring the VL image, until such subspecialty research has been undertaken, and guidelines produced at a local level to reflect this. A recent prospective observational study into the use of VL by the helicopter emergency medical service (HEMS) bases of the Swiss Air Rescue over the course of one year demonstrated an overall 87.6% first pass success (FPS) rate including 84.8% in trauma [21]. Of note in this study, performing airway management indoors or inside an ambulance resulted in a significantly higher FPS of 91.1% compared to outdoor locations. This increased success when not outside may be specific to VL however as it mitigates glare that can reduce clarity on the VL image. This choice of location inside/ in an ambulance must also be balanced against the numerous benefits of having 360 degree access to a patient when they are not located in an enclosed environment.

As with all procedures, operator choice and familiarity is imperative. In light of the lack of any strong evidence in favour of one particular method of gaining airway control, this personal preference and familiarity should continue to guide RSI technique choice.

Front of neck access

Should intubation be unsuccessful however, in a 'cannot intubate cannot ventilate scenario', Front of Neck Access (FONA) would likely be attempted. Again, the evidence for this in the pre-hospital PNI population is positive. A large prospective observational study based at combat hospitals in Iraq and Afghanistan identified 34 patients out of a total of 1927 who received a pre-hospital or in-transfer cricothyrotomy, an incidence of 1.76%. 83% of these patients had significant head, face and neck injuries. This incidence likely reflects the differences in support, resources and transfer durations between military and civilian systems. Cricothyrotomy was successful in 28 cases (82%) [22]. Of note in this paper all FONA was performed by non-physicians, reiterating that operator familiarity, competency, training and experience is more important than clinical background. This 82% success rate is slightly lower than the 90.5% reported in a previous meta-analysis [23], likely due to the more serious injury profile, and by extension anatomical disruption, in the military patient cohort. In comparison, a review of the last 20 years of experience of London HEMS demonstrated a much lower incidence of only 0.19% (72 of 37,725 patients) with a 97% success rate [24]. Of interest, penetrating trauma (stabbing of gunshot wound) at any anatomical location, accounted for only 8 (10.3%) cases of scalpel cricothyroidotomy over this 20-year case review.

In the scenario that definite airway control cannot be achieved through the above two routes, another option reported in the literature, and one to consider if applicable, is to use a bougie to facilitate intubation directly through a tracheal laceration/puncture [25].

Ventilation

Once a definitive airway has been obtained and ventilation commenced, it is important to be mindful of the chest complications of both the initial trauma and positive pressure ventilation. This is particularly important in zone 1 injuries with a caudal vector of penetration. Haemothorax and pneumothorax are reported as important causes of respiratory failure in PNI [26] and in an Israeli military report of neck injuries over a 20-year period, 2% of isolated neck injuries required chest decompression [27].

In conclusion, should definitive airway control be required, and clinicians present with the available skill set to perform an RSI, this should be conducted based on the operators familiarity and preference with a given technique.

Circulation

As with all trauma patients, initial circulatory resuscitation comprises ongoing haemorrhage control, haemostatic volume resuscitation and measures to prevent trauma coagulopathy. Haemostatic resuscitation and mitigation of trauma coagulopathy are beyond the scope of this review. Instead, we will focus on haemorrhage control measures specific to PNI.

The role of haemostatic dressings has already been evaluated. These can be augmented if required with other devices, one of which is the iTClamp® (Innovative

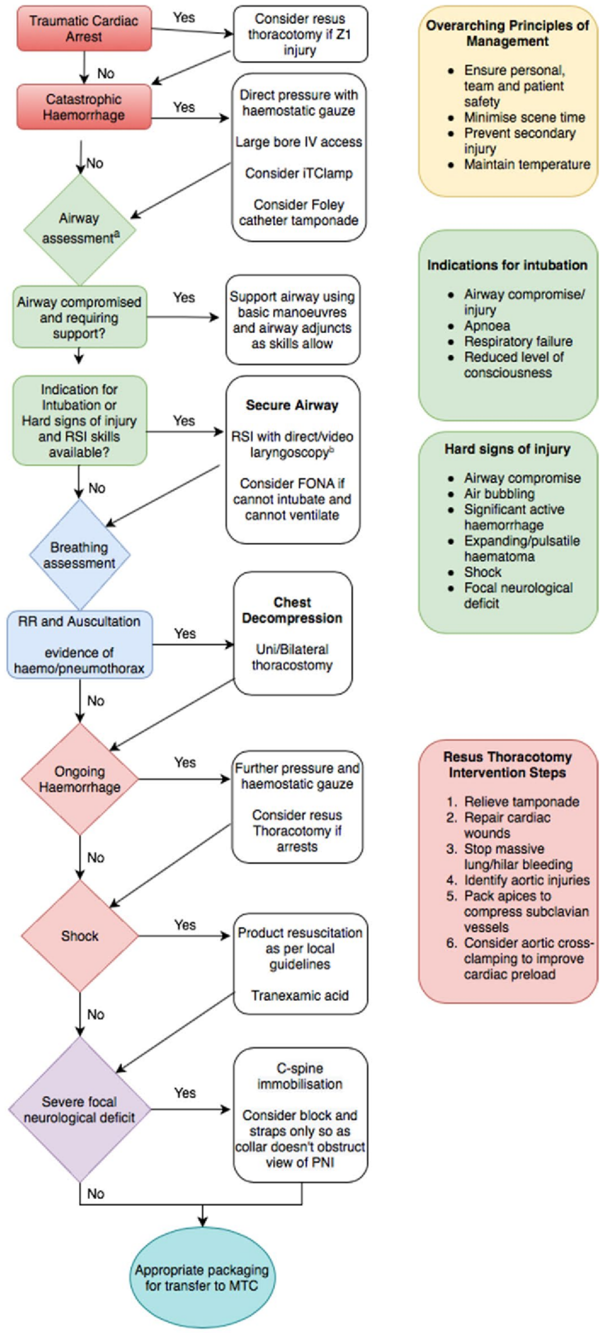

a) consider removing Manual InLine Stabilisation at this point if penetrating injury only and no evidence of severe focal neurological deficit
b) Choice of DL/VL should be based on operator familiarity and preference alongside individual service guidelines

Fig. 2 Proposed PNI management flow diagram

Trauma Care, San Antonio, TX, USA). This is a temporary wound closure device that can be applied to control haemorrhage from open wounds in compressible zones [28]. A case series of 10 patients, 50% of whom had mixed arterial and venous bleeding, showed 90% control of bleeding using this device. This was increased to 100% when the device and haemostatic gauze were used in combination [29]. This case series, along with its demonstration of effectiveness in over 245 field applications, has led to the iTClamp® being recommended as a primary intervention for haemorrhage control in PNI by the American committee on Tactical Combat-Casualty Care [30].

Foley catheters can also be used to manage haemorrhage in PNI. A small military case series demonstrated 11 patients in whom a Foley catheter was used to control haemorrhage with a 91% success rate [31]. The authors report that a volume of 10 ml is usually sufficient to arrest bleeding without affecting the surrounding microcirculation.

In PNI resulting in traumatic cardiac arrest, current guidelines from the Western Trauma Association indicate that patients with PNI, less than 5 min of CPR and profound refractory shock should undergo a resuscitative thoracotomy (RT) [32]. This RT is to evaluate and arrest/mitigate ongoing sources of haemorrhage where possible. It would also allow, if indicated, aortic cross-clamping to increase cardiac pre-load and by extension cerebral and cardiac perfusion. A single trauma center in the US reports 27 patients undergoing RT for non-torso injuries over a 26-year period. Three of these 27 patients (11%) survived to leave hospital of which 2 of the 3 had sustained a PNI [33].

In summary, alongside local haemostatic volume resuscitation and haemorrhage control guidelines, junctional injury specific measures should also be considered including adjuncts such as the iTClamp® or a Foley catheter. Resuscitative thoracotomy should also be considered if indicated.

Disability prevention

Nervous system injuries occur in only approximately 7% of cases of PNI [34] and this drops further to 1.5% when looking at stab wounds only [35]. These authors further concluded that unstable cervical spine injuries in PNI are very rare but are associated with severe focal neurological deficit or altered mental status. This sits alongside a previous retrospective review from the same time period that associated cervical spine immobilisation with an increased risk of death in PNI [36].

More recently Haut et al. [37] retrospectively analysed data from more than 45,000 patients with isolated penetrating trauma. The odds ratio for death in patients undergoing spinal stabilisation was 2.06 (95% CI 1.35–3.13) compared to those that did not. The NNT with spinal stabilisation to potentially benefit 1 patient was 1032, whereas the NNH was 66.

In ballistics injuries, including explosions and gunshot wounds, in a series of 90 British military casualties, only 1.8% of survivors to a surgical facility had sustained an unstable cervical spine injury that required surgical stabilisation [38].

Furthermore, a retrospective review of battlefield casualties including 44 cases of PNI in Israeli soldiers concluded that c-spine collars had the potential to hide signs of life-threatening conditions, including tracheal deviation, subcutaneous emphysema, large expanding haematoma, and diminished or absent carotid pulses [39].

The above evidence has led to the conclusion of a strong recommendation against the intervention of spinal stabilisation in patients with isolated penetrating injuries, in a recent update on spinal stabilisation in adult trauma patients [40].

Along with judicious use of c-spine immobilisation for patients with hard neurological signs, neuroprotective measures should be considered to prevent any secondary neurological injury. This includes, where possible, prevention of hypoxia, hypercapnia and hypotension.

Limitations of this review

This review has looked at an area of current practice within which, patient numbers are relatively small, but who can have significant morbidity and mortality. This is also an area of practice split between both civilian and military environments, within which practice, resources, and geography can be different. Finally, there is also a relative lack of evidence from pre-hospital practice due to a combination of its emergency nature, austere environment, challenges in performing prospective high-quality trials, especially for interventions such as these, and the relatively recent emergence of pre-hospital emergency medicine as a specialty in itself. The combination of the above necessitates the use of more low-level evidence, predominantly case-series, and evidence extrapolated from alternative populations, to guide the recommendations made in this review. Given that pre-hospital practice recommendations are sometimes extrapolated from in-hospital research papers, a search focusing on pre-hospital papers only could also be deemed a limitation.

This review also only evaluated those papers written in English language. As such there is also the potential that other relevant research may have been omitted.

Discussion

PNI is a relatively rare yet clinically challenging presentation faced by the pre-hospital clinical team. Evidence in this field is mainly comprised of case series. We identified only one meta-analysis and one randomized controlled trial. It is unlikely that a prospective randomised controlled trial will be preformed in this area, therefore an expert consensus statement on best practice, derived from the best available evidence, should be used to guide clinical practice. The use of a shared structured mental model including some nuanced additions as discussed in this review, may allow for rapid assessment and management prior to transfer to definitive care at a trauma centre. We have extrapolated from the results of this literature review, a proposed management algorithm for use in the pre-hospital environment, when managing PNI (Fig. 2). Interventions commonly employed in the pre-hospital setting, such as RSI for definitive airway control, should continue to be used, as should basic haemorrhage control interventions including direct pressure with haemostatic gauze. Cervical spine immobilisation should be avoided in all cases of PNI unless there is evidence of severe focal neurological deficit or altered mental status.

Future research in this field should now seek to differentiate best practice within this model in more detail, using studies to provide a higher level of evidence than that which is currently available. This may include direct vs. video laryngoscopy in PNI, or between different types of haemostatic gauze and other immediate haemorrhage control interventions. The effects of these interventions on preventing secondary brain injury should also be evaluated.

Conclusion

Penetrating neck injury is a rare presentation yet one which is clinically challenging and has high patient mortality and morbidity. This review leads to a proposed algorithm for the pre-hospital management of penetrating neck injuries, based on current available evidence. On this algorithm we hope to base an onward Delphi process to agree a consensus statement to guide the management of PNI in the civilian population.

Abbreviations

PNI: Penetrating neck injury; RSI: Rapid sequence induction; FONA: Front of neck access; VL: Video laryngoscopy; DL: Direct laryngoscopy; NNT: Number needed to treat; NNH: Number needed to harm; HEMS: Helicopter emergency medical services.

Acknowledgements

The authors would like to acknowledge Zane Perkins for his assistance in the proposed Resus Thoracotomy intervention steps within figure 2.

Authors' contributions

CS was the primary author of the manuscript. HT and AH were significant contributors to the manuscript. All authors read and approved the final manuscript.

Funding

The authors are in receipt of no funding pertaining to this topic or article.

Availability of data and materials

Not applicable, review article.

Declarations

Ethics approval and consent to participate

Not applicable.

Consent for publication

Not applicable.

Competing interests

There are no competing interests to be declared.

Author details

[1]Emergency Department, St. George's Hospital Trust, Blackshaw Rd., Tooting, London SW17 0QT, UK. [2]Air Ambulance Kent Surrey Sussex, Redhill Airfield, Redhill RH1 5YP, Surrey, UK.

Received: 7 June 2021 Accepted: 2 September 2021
Published online: 16 September 2021

References

1. Brywczynski J, Barrett T, Lyon J, Cotton B. Management of penetrating neck injury in the emergency departemtn: a structured literature review. Emerg Med J. 2008;25:711–5.
2. Sperry J, Moore E, Coimbra R. Western Trauma Association critical decisions in trauma: penetrating neck trauma. J Trauma Acute Care Surg. 2013;75:936–40.
3. Roon A, Christensen N. Evaluation and treatment of penetrating injuries of the neck. J Trauma. 1979;19:391–4.
4. Low G, Inaba K, Chouliaras K. The use of the anatomic "Zones" of the neck in the assessment of penetrating neck injury. Am Surg. 2014;80:970–4.
5. Inaba K, Branco B, Menaker J. Evaluation of multidetector computed tomography for penetrating neck injury: a prospective multicenter study. J Trauma Acute Care Surg. 2012;72:576–83.
6. Biffl W, Moore E, Rehse D. Selective management of penetrating neck trauma based on cervical level of injury. Am J Surg. 1997;174:678–82.
7. Demetriades D, Theodorou D, Cornwell E. Evaluation of penetrating injuries of the neck: prospective study of 223 patients. World J Surg. 1997;21:41–7.
8. Osborn T, Bell R, Qaisi W, Long W. Computed tomographic angiography as an aid to clinical decision making in the selective management of penetrating injuries of the neck: a reduction in the need for operative exploration. J Trauma. 2008;64:1466–72.
9. van Oostendorp S, Tan E, Geeraedts L. Pre-hospital control of life-threatening truncal and junctional haemorrhage is the ultimate challenge in optimizing trauma care; a review of treatment options and their applicability in the civilian trauma setting. Scand J Trauma Resusc Emerg Med. 2016;24:110.
10. Boulton A, Lewis C, Naumann D, Midwinter M. Prehospital haemostatic dressings for trauma: a systematic review. Emerg Med J. 2018;35:449–57.
11. Shina A, Lipsky A, Nadler R, et al. Prehospital use of hemostatic dressings by the Israel Defense Forces Medical Corps: a case series of 122 patients. J Trauma Acute Care Surg. 2015;79(4):S204–9.
12. Ministry of Defense. Clinical guidelines for operation. London: Joint Service Publication; 2013. p. 999.

13. Hatamabadi H, Asayesh Z, Kariman H, et al. Celox-coated gauze for the treatment of civilian penetrating trauma: a randomized clinical trial. Trauma Mon. 2015;20(1):e23862.
14. Leonard J, Zietlow J, Morris D, et al. A multi-institutional study of haemostatic gauze and tourniquets in rural civilian trauma. J Trauma Acute Care Surg. 2016;81:441–4.
15. Kristensen M, McGuire B. Managing and securing the bleeding upper airway: a narrative review. Can J Anesth. 2020;67:128–40.
16. Crewdson K, Lockey D, Voelckel W, et al. Best practice advice on pre-hospital emergency anaesthesia and advanced airway management. Scand J Trauma Resusc Emerg Med. 2019. https://doi.org/10.1186/s13049-018-0554-6.
17. Mercer S, Jones C, Bridge M, et al. Systematic review of the anaesthetic management of non-iatrogenic acute adult airway trauma. Br J Anaesth. 2016;117(Suppl 1):i49–59.
18. Mandavia D, Qualls S, Rokos I. Emergency airway management in penetrating neck injury. Ann Emerg Med. 2000;35:221–5.
19. Shearer V, Giesecke A. Airway management for patients with penetrating neck trauma: a retrospective study. Anesth Analg. 1993;77:1135–8.
20. Lewis S, Butler A, Parker J, Cook T, Smith A. Videolaryngoscopy versus direct laryngoscopy for adult patients requiring tracheal intubation. Cochrane Database Syst Rev. 2016;11(11):CD011136.
21. Knapp J, Eberle B, Bernhard M, et al. Analysis of tracheal intubation in out-of-hospital helicopter emergency medicine recorded by video laryngoscopy. Scand J Trauma Resusc Emerg Med. 2021;29:1–10.
22. Barnard E, Ervin A, Mabry R, Bebarta V. Prehospital and en route cricothyotomy performed in the combat setting: a prospective, multicentre, observational study. J Spec Oper Med. 2014;14:35–9.
23. Hubble M, Wilfong D, Brown L, Hertelendy A, Benner R. A meta-analysis of prehospital airway control techniques part II: alternative airway devices and cricothyrotomy success rates. Prehosp Emerg Care. 2010;14:515–30.
24. Aziz S, Foster E, Lockey D, Christian M. Emergency scalpel cricothyroidotomy use in a prehospital trauma service: a 20-year review. Emerg Med J. 2021;38:349–54.
25. Battacharya P, Mandal M, Das S. Airway management of two patients with penetrating neck trauma. Indian J Anaesth. 2009;53:348–51.
26. Hanlon D, Adams D. Penetrating neck trauma. *Trauma Rep.* 2017.
27. Tsur N, Benov A, Nadler R, et al. Neck Injuries-Israel defense forces 20 years' experience. Injury. 2020;52(2):274–80.
28. Filips D, Logsetty S, Tan J, et al. The iTClamp controls junctional bleeding in a lethal swine exanguination model. Prehosp Emerg Care. 2013;17:526–32.
29. Tan E, Peters J, Mckee J, Edwards M. The iTClamp in the management of prehospital haemorrhage. Injury. 2016;47:1012–5.
30. Onifer D, Mckee J, Fudree L, et al. Management of haemorrhage from craniomaxillofacial injuries and penetrating neck injury in tctical combat casualty care: iTClamp mechanical wound closure device TCCC guidelines proposed change June 2019. J Spec Oper Med. 2019;19(3):31–44.
31. Jose A, Arya S, Nagori S, Thukral H. Managment of life-threatening haemorrhage from maxillofacial firearm injuries using Foley catheter balloon tamponade. Craniomaxillofac Trauma Reconstr. 2019;12:301–4.
32. Burlew C, Moore E, Moore F, et al. Western trauma association critical decisions in trauma: resuscitative thoracotomy. J Trauma Acute Care Surg. 2012;73:1359–63.
33. Sheppard F, Cothren C, Moore E, et al. Emergency department resuscitative thoracotomy for nontorso injuries. Surgery. 2006;139:574–6.
34. Vanderlan W, Tew B, Seguin C, et al. Neurolgogic sequelae of penetrating cervical trauma. Spine. 2009;34:2646–53.
35. Lustenberger T, Talving P, Lam L, et al. Unstable cervical spine fracture after penetrating neck injury: a rare entity in an analysis of 1069 patients. J Trauma. 2011;70(4):870–2.
36. Vanderlan W, Tew B, Mcswain N Jr. Increased risk of death with cervical spine immobilisation in penetrating cervical trauma. Injury. 2009;40:880–3.
37. Haut E, Kalish B, Efron D, et al. Spine immobilisation in penetrating trauma: more harm than good? J Trauma Injury Infect Crit Care. 2010;68:115–21.
38. Ramasamy M, Midwinter M, Mahoney P, Clasper J. Learning the lessons from conflict: pre-hospital cervical spine stabilisation following ballistic neck trauma. Injury. 2009;40:1342–5.
39. Barkana Y, Stein M, Scope A, et al. Prehospital stabilization of the cervical spine for penetrating injuries of the neck—is it necessary? Injury. 2000;31(5):305–9.
40. Maschmann C, Jeppesen E, Rubin M, Barfod C. New clinical guidelines on the spinal stabilisation of adult trauma patients-consensus and evidence based. Scand J Trauma Resusc Emerg Med. 2019;27:77.

Publisher's Note

Springer Nature remains neutral with regard to jurisdictional claims in published maps and institutional affiliations.

Ready to submit your research? Choose BMC and benefit from:

- fast, convenient online submission
- thorough peer review by experienced researchers in your field
- rapid publication on acceptance
- support for research data, including large and complex data types
- gold Open Access which fosters wider collaboration and increased citations
- maximum visibility for your research: over 100M website views per year

At BMC, research is always in progress.

Learn more biomedcentral.com/submissions

BMC

Read the scoping review here

[Attachment: "Penetrating Neck Injury Scoping Review_Simpson et al.pdf"]

Were the search criteria used in the scoping review robust?
◯ Yes
◯ No

If no, please outline your concerns below

____

Is there any evidence or papers that are not discussed in the scoping review that you would expect?
◯ Yes
◯ No

If yes, please outline below

____

In the scoping review, regarding the initial assessment of the patient, are you in agreement with the list of hard signs (massive sub-cutaneous emphysema/ air bubbling, airway compromise, expanding or pulsatile haematoma, active bleeding, shock, haematemesis/ haemoptysis, neurologic deficit)?
◯ Yes
◯ No

If no, please outline your recommendations

____

In the scoping review, are you in agreement with the list of soft signs (proximity of wound to the carotid artery/jugular vein, non-expanding haematoma, change of voice/hoarseness, dysphagia, upper extremity pulse deficit, palpable crepitus)?
◯ Yes
◯ No

If no, please outline your recommendations

____

Catastrophic haemorrhage In relation to PNI, please indicate your agreement with the following statements

0 - strongly disagree
10 - strongly agree

Packing of wounds with haemostatic gauze (when possible) and application of direct pressure represents the mainstay of treatment

0 5 10

(Place a mark on the scale above)

For wounds that are not able to be managed with packing and pressure consideration should be given to the use of the ITClampTM

0 5 10

(Place a mark on the scale above)

An ITClamp should be considered prior to haemostatic gauze and pressure as the principle method of haemorrhage control

0 5 10

(Place a mark on the scale above)

When haemorrhage cannot be controlled with either haemostatic gauze and pressure or an ITClamp a Foley catheter should be inserted into the wound and inflated

0 5 10

(Place a mark on the scale above)

Once haemostasis has been achieved the dressing or other device should not be removed until a place of safety is reached i.e. hospital with appropriate surgical skills to control haemorrhage

0 5 10

(Place a mark on the scale above)

Please make any comments on catastrophic haemorrhage below

____

Airway and ventilation In relation to PNI, please indicate your level of agreement with the following indications for intubation

0 - strongly disagree
10 - strongly agree

Airway compromise

0 5 10

(Place a mark on the scale above)

Airway injury

0 5 10

(Place a mark on the scale above)

Apnoea

0 5 10

(Place a mark on the scale above)

Respiratory failure

0 5 10

(Place a mark on the scale above)

Hypoxia

0 5 10

(Place a mark on the scale above)

Reduced level of conciousness

0 5 10

(Place a mark on the scale above)

Other than the 6 indications above, do you feel there are any missing indications for emergency intubation?

____

With relation to airway and ventilation in the patient with a PNI, how much do you agree with the following statements?

0 - strongly disagree
10 - strongly agree

Statement	Scale
Whenever possible spontaneous ventilation should be maintained	0 — 5 — 10 *(Place a mark on the scale above)*
The first attempted intervention should be RSI	0 — 5 — 10 *(Place a mark on the scale above)*
Cricoid pressure should be avoided	0 — 5 — 10 *(Place a mark on the scale above)*
Positive pressure ventilation via a facemask should be avoided	0 — 5 — 10 *(Place a mark on the scale above)*
Positive pressure ventilation via a supraglottic airway should be avoided	0 — 5 — 10 *(Place a mark on the scale above)*
Video laryngoscopy is favoured over direct laryngoscopy	0 — 5 — 10 *(Place a mark on the scale above)*
Choice of laryngoscopy device should be guided by experience and personal preference	0 — 5 — 10 *(Place a mark on the scale above)*
Front of neck access should be attempted in a cannot intubate cannot ventilate scenario	0 — 5 — 10 *(Place a mark on the scale above)*
If a definitive airway cannot be achieved through RSI, direct intubation of a transected trachea should be considered if applicable	0 — 5 — 10 *(Place a mark on the scale above)*

Please detail any comments on airway and ventilation

____

Resuscitation measures In the context of cardiac arrest resulting from penetrating neck injury resuscitative thoracotomy should be considered if the appropriate skill mix is present for patients who have sustained injury to the following zone of the neck:

0 - strongly disagree
10 - strongly agree

Zone 1 — 0 … 5 … 10

(Place a mark on the scale above)

Zone 2 — 0 … 5 … 10

(Place a mark on the scale above)

Zone 3 — 0 … 5 … 10

(Place a mark on the scale above)

In the context of cardiac arrest resulting from penetrating neck injury resuscitative thoracotomy should be considered if the appropriate skill mix is present for patients in which time frame?

e.g. A score of 5 would equate to after 5 minutes of haemorrhage control and maximal resuscitation.

0 - Immediately
20 - 20 minutes

0 … 10 … 20

(Place a mark on the scale above)

Please detail any comments on resuscitative measures

____

In the context of cardiac arrest resulting from penetrating neck injury please assign number to the following interventions in order of priority

Airway management

- ◯ 1 - top priority
- ◯ 2 - second priority
- ◯ 3 - third priority
- ◯ 4 - lowest priority

Haemorrhage control

- ◯ top priority
- ◯ second priority
- ◯ third priority
- ◯ lowest priority

Vascular access and volume resuscitation

- ◯ top priority
- ◯ second priority
- ◯ third priority
- ◯ lowest priority

Thoracotomy

◯ top priority
◯ second priority
◯ third priority
◯ lowest priority

Disability In relation to PNI, please indicate your level of agreement with the following statements

0 - strongly disagree
10 - strongly agree

Cervical spine control should be reserved for patients with hard neurological signs

0 5 10

(Place a mark on the scale above)

Cervical spine control should be applied in those patients with a reduced consciousness level

0 5 10

(Place a mark on the scale above)

Cervical spine control should be applied to those patients who have sustained a gun shot wound to the neck

0 5 10

(Place a mark on the scale above)

Cervical collars should be avoided and spinal immobilisation achieved through the use of alternative methods such as head blocks whenever possible

0 5 10

(Place a mark on the scale above)

Cervical spine control should be applied to those patients who have sustained a wound to the neck following a blast injury

0 5 10

(Place a mark on the scale above)

Please list any comments on spinal immobilisation including any additional indications

____

Review of the proposed algorithm

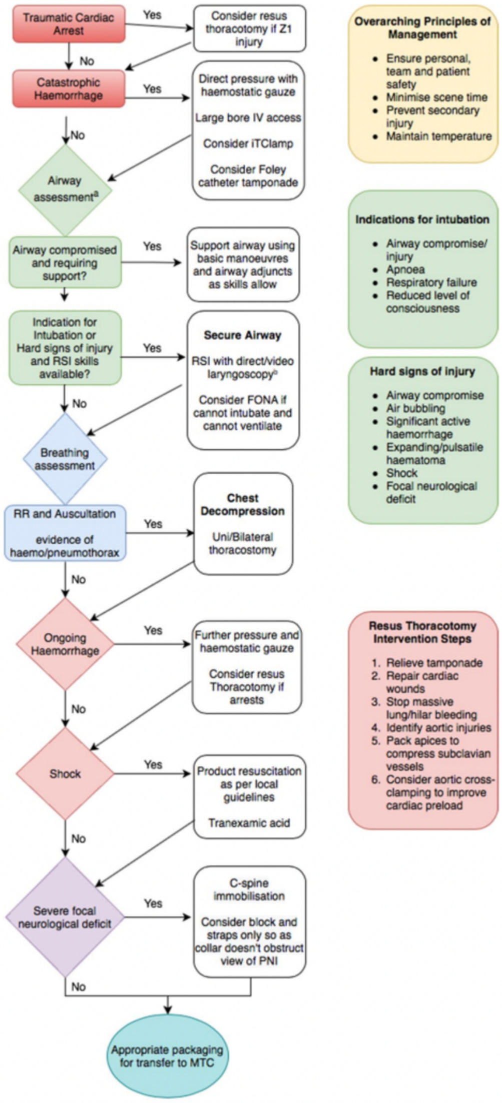

a) consider removing Manual InLine Stabilisation at this point if penetrating injury only and no evidence of severe focal neurological deficit
b) Choice of DL/VL should be based on operator familiarity and preference alongside individual service guidelines

Fig. 2 Proposed PNI management flow diagram

Do you agree with the overarching principles of the PNI management algorithm?	◯ Yes ◯ No
Please list any concerns or suggestions you have with respect to the proposed algorithm and document any proposed changes you would make to the algorithm	__________________________________
Do you agree with the steps listed during a resuscitative thoracotomy?	◯ Yes ◯ No
Please list any concerns or suggestions you have with respect to resuscitative thoracotomy	__________________________________
Do you agree with the proposed indications for chest decompression (clinical evidence of respiratory compromise and signs of haemo/pneumothorax)?	◯ Yes ◯ No
If no, what other indications would you include for chest decompression?	__________________________________

Thank you for taking the time to complete our survey
